# Impact of *FMR1* Premutation on Neurobehavior and Bioenergetics in Young Monozygotic Twins

**DOI:** 10.3389/fgene.2018.00338

**Published:** 2018-08-27

**Authors:** Eleonora Napoli, Andrea Schneider, Randi Hagerman, Gyu Song, Sarah Wong, Flora Tassone, Cecilia Giulivi

**Affiliations:** ^1^Department of Molecular Biosciences, School of Veterinary Medicine, University of California, Davis, Davis, CA, United States; ^2^UC Davis MIND Institute, UC Davis Health, Sacramento, CA, United States; ^3^Department of Pediatrics, School of Medicine, University of California, Davis, Sacramento, CA, United States; ^4^Department of Biochemistry and Molecular Medicine, School of Medicine, University of California, Davis, Sacramento, CA, United States

**Keywords:** bioenergetics, FXTAS, mitochondrial dysfunction, oxidative stress, premutation

## Abstract

Mitochondrial dysfunction (MD) has been identified in lymphocytes, fibroblasts and brain samples from adults carrying a 55–200 CGG expansion in the fragile X mental retardation 1 (*FMR1*) gene (premutation; PM); however, limited data are available on the bioenergetics of pediatric carriers. Here we discuss a case report of three PM carriers: two monozygotic twins (aged 8 years) harboring an *FMR1* allele with 150–180 CGG repeats, with no cognitive or intellectual issues but diagnosed with depression, mood instability and ADHD, and their mother (asymptomatic carrier with 78 CGG repeats). Fibroblasts and lymphocytes from the twins presented a generalized OXPHOS deficit, altered mitochondrial network, accumulation of depolarized mitochondria, and increased mitochondrial ROS production, outcomes distinct and more severe than the mother’s ones, suggesting the involvement of modulatory effects mediated by CGG expansion, X-activation ratio, sex hormones and epigenetic factors (chronic inflammation, consequence of Lyme disease). The degree of the severity of MD appeared to segregate with the morbidity of the phenotype. The mitochondrial ROS-mediated HIF-1α stabilization was identified as a key player at contributing to the MD, pointing it as a novel target for future therapeutical intervention.

## Introduction

Premutation (PM) carriers are individuals with a modestly expanded CGG nucleotide repeat (55–200) in the 5′-UTR of the fragile X mental retardation gene, *FMR1* (Hagerman and Hagerman, 2013). Adult carriers often have a subtle phenotypic profile, worsening as they age into poor performance on executive and visuospatial tasks, increased difficulties with math and increased symptoms of anxiety. The PM in adults increases the risk for developing the neurodegenerative disease known as FXTAS (Berry-Kravis et al., 2007) and, in women only, FXPOI (Sherman, 2000).

Pediatric PM carriers (≤18 years old) are often diagnosed with ADHD, anxiety and other psychopathologies (Bailey et al., 2008), albeit at a lower incidence than in individuals affected with FXS, the most common inherited form of intellectual disability, carrying a full *FMR1* mutation (>200 CGG repeats). Indeed, the incidence of ASD in PM carriers is ∼15% (Farzin et al., 2006) whereas in FXS is 60% (Hagerman et al., 2010). In the first national parent-report survey of families with FXS performed with a relatively large sample size (Bailey et al., 2008) parents reported higher frequencies of developmental delay (boys and girls), attention problems (boys and girls), aggressiveness (boys), autism (boys), seizures (boys), anxiety (boys and girls) and depression (girls) in pediatric carriers. Two other studies found developmental differences in infants carrying the PM compared with NC controls, differences that may be viewed as early markers of anxiety, social deficits, or other developmental challenges later in life (Wheeler et al., 2016).

Bioenergetic deficits, or mitochondrial dysfunction (MD), with increased oxidative stress biomarkers have been observed in post-mortem brain samples (Ross-Inta et al., 2010), lymphocytes (Napoli et al., 2016a), fibroblasts (Ross-Inta et al., 2010; Napoli et al., 2016b), and plasma (Giulivi et al., 2016) from young and adult PM carriers. These deficits correlated with both CGG repeat expansion and severity of the phenotype (Ross-Inta et al., 2010; Napoli et al., 2011). Given the critical role of mitochondria in energy and neurotransmitter metabolism (Laughlin et al., 1998), MD may contribute to the adult PM phenotype, but whether these metabolic changes appear at early stages of life is unknown.

In this study, we present a case of MZ twins identified as PM carriers (males, aged 8 years), recruited through the Fragile X Treatment and Research Center (MIND Institute at UC Davis). They exhibited relatively long and unstable CGG repeat (upper PM range) in the 5′UTR of the *FMR1* gene in both fibroblasts and lymphocytes and were diagnosed with severe psychological and emotional problems but no cognitive deficits. MZ twins have identical genotypes, and any putative differences in outcomes are theoretically due to environmental factors. Thus, the aim of this study was to investigate the impact of genetic versus non-genetic factors by evaluating the bioenergetics of lymphocytes and primary dermal fibroblasts from young MZ PM carriers and their mother (a relatively asymptomatic PM carrier) compared to age-matched NC, and to determine whether the putative MD segregated with either specific symptoms and/or phenotypic severity.

## Materials and Methods

### Chemicals and Biochemicals

EDTA, EGTA, KH_2_PO_4,_ sodium succinate, digitonin, rotenone, antimycin A, oligomycin, malonate, ascorbic acid, *N,N,N′,N′-*tetramethyl-*p-*phenylenediamine, KCN and HEPES were purchased from Sigma (St Louis, MO, United States). Tris–HCl, glycine, sodium chloride and KCl were purchased from Fisher (Pittsburg, PA, United States). Bovine serum albumin (fatty acid free) was obtained from MP Biomedicals. All reagents were of analytical grade or higher.

### Isolation of Lymphocytes From Blood Samples and Fibroblasts Growing Conditions

Blood samples were obtained from both twins at three different times (August 2013, January 2014, and March 2016). Blood samples from both parents were collected only at the latest time point. All blood samples (including the ones obtained from adult donors, average age ± SEM = 37.4 ± 3.5) were collected by venipuncture with informed consent at the MIND Institute and approved by the institutional review board ethics committee at UC Davis Medical Center. Blood (5–8 ml) was collected in Vacutainer CPT tubes (Applied Biosystems, Foster City, CA, United States) and lymphocytes were isolated as previously described (Napoli et al., 2016a).

Skin biopsies from the twins and their mother were obtained in January 2014. To minimize confounding factors, our study was focused in several ways: (i) We concentrated on bioenergetics deficits that were conserved in primary (lymphocytes) and primary culture of skin fibroblasts. (ii) All fibroblasts were studied at the same early stage of population doubling (passages 6–10), where significant telomere shortening and corresponding features of replicative senescence could be excluded. (iii) The study was restricted to dermal fibroblasts derived from age- and sex- matched healthy adult donors for the mother and the twins, thus excluding age or gender influence on skin aging. (iv) Skin fibroblasts were isolated from the same skin area (punch biopsy on left upper back) to minimize variances due to body site or different exposure to the external milieu. All fibroblasts from carriers were obtained from Dr. P. Hagerman. Age- and sex-matched fibroblast controls were obtained from the Coriell Biorepository (Camden, NJ, United States). Fibroblasts were grown in high glucose Minimum Essential Medium supplemented with 15% FBS, 2 mM glutamine, 1 mM sodium pyruvate, as previously described (Napoli et al., 2011).

### Isolation of Genomic DNA, CGG Sizing and qRT-PCR

Genomic DNA was isolated from lymphocytes using Gentra Puregene Blood Kit (Qiagen, Valencia, CA, United States). CGG sizing was obtained using a combination of PCR (AmplideX PCR/CE FMR1 Kit; Asuragen Inc. Austin, TX) and Southern blot analysis (Tassone et al., 2008) and measured as previously described (Filipovic-Sadic et al., 2010). Methylation status of the *FMR1* promoter and specifically the XAR in the mother and the percent of methylation in the two twins was measured on Southern Blot using the Alpha Innotech FluorChem 8800 Image (Tassone et al., 1999). Total RNA was obtained from whole blood collected in Tempus tubes using manufacture instructions (Applied Biosystems, Foster City, CA, United States) and qRT-PCR was performed as detailed in Tassone et al. (2000).

### Evaluation of Mitochondrial Mass, Morphology and Distribution by Confocal Microscopy in Fibroblasts

Cells (passages 7–10, 1 × 10^5^) were seeded on sterile coverslips, grown over night at 37°C and then incubated for 30 min at 37°C with 0.5 μM MitoTracker Red CMXRos (MolecularProbes Inc., Eugene, OR, United States) diluted in growth media (Napoli et al., 2016b). More details are provided under **Supplementary Information**. For mitochondrial morphology quantification, images were further analyzed with two macro tools designed for ImageJ (Fiji) (Dagda et al., 2009; Valente et al., 2017) to allow the quantification of mitochondrial mass, morphological features and network integrity. Mitochondrial cellular distribution was evaluated using the Fiji surface plot feature, followed by plot profile analysis.

### Mitochondrial Bioenergetics

Mitochondria-dependent oxygen consumption was evaluated in either fibroblasts or freshly isolated lymphocytes permeabilized with digitonin as previously described (Giulivi et al., 2010). Methodological details are reported under **Supplementary Information**. Citrate synthase activities were evaluated spectrophotometrically as described elsewhere (Napoli et al., 2011) using the equivalent of 2.0–5.0 × 10^4^ cells.

### Mitochondrial ROS Production

Cells (passages 7–10, 1 × 10^5^) were seeded on sterile cover slips, grown over night at 37°C and subsequently incubated for 10 min at 37°C with 5 μM MitoSOX (ThermoFisher Scientific), prepared as per manufacturer’s instruction and diluted in growth media, washed thoroughly and subsequently counterstained with 1 μg/ml 4′,6 diamidino-2-phenylindole (DAPI) and mounted on glass slides with ProLong Gold anti-fade mounting medium for fixed cells. Images (10–15 for each cell line) were obtained with an Olympus FV1000 laser scanning confocal microscope (excitation and emission wavelengths 594 and 660 nm) at 60× magnification. Analysis of fluorescence intensity was carried out with Fiji and normalized by cell area.

### Gene Expression in Fibroblasts From Premutation Carriers and Age-Matched Controls

RNA was isolated from fresh fibroblast cells containing 2 × 10^6^ cells using the RNEasy Plus Mini Kit from Qiagen (cat. no. 74134) following the manufacturer’s instructions. cDNA was synthesized using Qiagen’s Quantitect RT kit (cat no. 205311) following manufacturers recommendations. RNA and cDNA concentrations were determined using the Tecan Infinite M200 Nanoquant plate reader (Tecan, Austria). All primers/probe mix were from Life Technologies (Grand Island, NY, United States). Sequences of commercial primers and probes are proprietary. cDNA was diluted to 40 ng/μl and served as stock template for qRT-PCR. See **Supplementary Information and Supplementary Figure 1** for more details.

### Evaluation of mtDNA Copy Number and Deletions

These assays were performed on genomic DNA extracted from lymphocytes and fibroblasts by using qRT-PCR and essentially as described in Giulivi et al. (2010). Other details are included under the **Supplementary Information**.

### Western Blotting

Fibroblasts from twins, their mother and relative controls were lysed in RIPA buffer and assessed by Western blots as previously described (Napoli et al., 2013). Membranes were visualized with the use of the Odyssey Infrared Imaging System (LI-COR). Densitometry analysis was carried out with ImageJ. The complete list of antibodies used with relative dilutions and commercial source is reported under **Supplementary Table 1**. Additional methodological details are under **Supplementary Methods**.

### Statistical Analysis

Data from twins’ and their mother are expressed as mean ± SD and compared to the 95% CI built with values from age- and sex-matched controls. The number of control samples (fibroblasts and lymphocytes) used to calculate the 95% CI for each outcome are reported in each of the individual tables and figures for both children and adults.

## Results

### Clinical Description of Carriers Presented in This Study

Both twins presented normal or above IQ with no intellectual or cognitive issues. However, both twins were diagnosed with depression, mood instability and ADHD. Twin 2 only was diagnosed with bipolar disorder, manic behavior, motor coordination problems, poor stamina, and chronic fatigue, the latter probably developed after an episode of Lyme disease (**Table 1** and **Supplementary Information**). The mother was a 38-year PM carrier (CGG repeats allele sizes: 43, 78), with no psychiatric or medical problems associated with the PM, but at age 36, she met criteria for FXPOI, reported some word retrieval problems and monthly migraines (more details under **Supplementary Information**).

**Table 1:** Clinical and medication history of Pre twins analyzed in this study.

	Twin 1	Twin 2
	**Developmental milestones (months)**	
Sitting independently (ref. 3–8 m)	6	6
Crawling (ref. 5–13 m)	10	9
Walking (ref. 8–18 m)	15	14
First words (ref. 12–18 m)	24	24
First sentences (ref. 24 m)	42	42

	**Characteristics at the time of examination**	

Blood pressure [mmHg [percentile] (National High Blood Pressure Education Program Working Group on High Blood Pressure in Children and Adolescents, 2004)]	88/56 [<50th]	93/50 [50th]
Head circumference [cm [percentile] (Rollins et al., 2010)]	52.5 [50th]	52.5 [50th]
Height [cm [percentile] (U.S. Department of Health and Human Services, 2008]	118.1 [15th ]	119.9 [19.6th]
Weight [kg [percentile] (U.S. Department of Health and Human Services, 2008]	18.96 [3.8th]	22.5 [34th]
Body Mass Index [percentile] (Kuczmarski et al., 2002)	13.6 [3rd]	15.6 [47th]
Full scale IQ (Wechsler, 2003)	123 [94th]	99 [47th]

	**Medications**	

Lisdexamfetamine (Vyvanse^TM^) (past)	Yes	No
Risperidone (Risperdal^TM^) (past)	No	Yes (0.5 mg)
Methylphenidate (Concerta^TM^) (current)	Yes	Yes (18 mg)
Guanfacine (Tenex^TM^) (current)	Yes	Yes
Sertraline (Zoloft^TM^) (current)	Yes (50 mg)	Yes (25 mg)
Melatonin (current)	Yes	No
*N*-acetyl-L-cysteine (current)	Yes (250 mg)	Yes (300 mg)
Vitamin B complex (current)	Yes	Yes
Omega 3s (current)	Yes	Yes
Aripiprazole (Abilify^TM^) (current)	Yes	Yes

	**Behavioral traits^∗^**	

	• Major depressive episode (age 5) in partial remission • Agoraphobia without history of panic disorder (age 5–7) in partial remission, specific phobia (animal type). • ADHD combined type • Eating disorder NOS in full remission. • Sub-threshold evidence of enuresis (not full clinical criteria)	• Depressive disorder NOS (age 7) in partial remission • Bipolar disorder NOS • ADHD combined type • Evidence of mania current and past • Motor coordination problems • Poor stamina after Lyme disease

	**Medical history**	

	• Recurrent ear infections • Inguinal hernia repair (age 3.5) • Reactive airway disease intermittently treated with a bronchodilator inhaler and occasional use of oral steroids and more frequent use of nasal steroids	• Recurrent ear infections • Umbilical hernia resolved in the first few years of life and recent repair of a right inguinal hernia • Reactive airway disease and seasonal allergies • Lyme disease (age 6) with erythema under his arms

NOS, Not Otherwise Specified; ADHD, Attention Deficit Hyperactivity Disorder; ^∗^diagnosis criteria for behavioral traits = KSADS or the Kiddie-schedule for affective disorders and schizophrenia. Developmental milestones normative values as well as other normative values were obtained from (National Research Council and Institute of Medicine, 2000; Wechsler, 2003; WHO Multicentre Growth Reference Study Group, 2006).

### Description of FMR1-Related Molecular Outcomes in Fibroblasts and Lymphocytes

**FIGURE 1:**
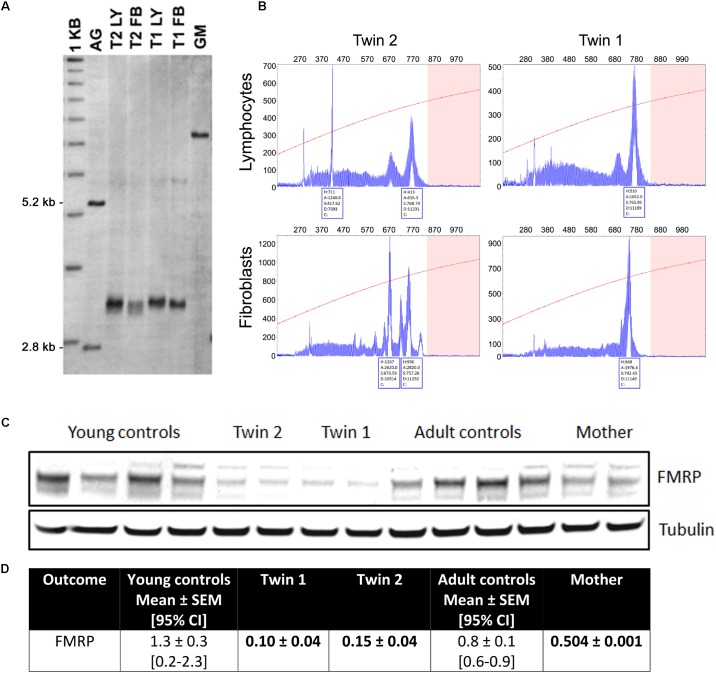
Length of the CGG repeat and methylation level in lymphocytes and fibroblasts from twins. **(A)** Representative Southern blot of CGG repeats carried out with lymphocytes (LY) and fibroblasts (FB) from twin 1 (T1) and twin 2 (T2). AG (lane 2) and GM (lane 7) are negative (non-carrier female) and positive (full mutation controls), respectively. Normal, unmethylated band (2.8 kbp) and normal, methylated band (5.2 kbp) are indicated on the left. Size marker (labeled as 1 kbp) is shown in Lane 1. **(B)** Capillary electrophoresis of PCR products amplified from genomic DNA isolated from blood and correspondent fibroblasts from the two twins demonstrates the presence of allele instability and somatic mosaicism in both blood and fibroblasts and in both subjects, as visualized by a series of peaks. The *x*-axis indicates the number of base pairs and the *y*-axis indicates relative fluorescence intensity**. (C)** Representative Western blot showing FMRP protein levels in fibroblasts from twins, their mother and correspondent age- and sex-matched controls (*n* = 4 for each sex- and age-matched group). Tubulin was used as loading control. **(D)** Densitometry values of FMRP normalized by tubulin intensity. 95% CI are shown in brackets. Data for the trio are shown as mean ± SD (as means of showing intra-experiment variability). Bolded are values lower than the 95% CI lowest limit.

Lymphocytes from the twins harbored *FMR1* alleles in the upper PM range of approximately the same size (157–180 CGG repeats for twin 1 and 150–180 for twin 2). Although a full mutation allele was not observed in the twins, we cannot exclude its presence in a very small percent of cells that was under the detection limit. In fibroblasts, the CGG repeats were approximately the same size as in the lymphocytes, as observed by Southern blots (**Figure 1A**). A broader range of CGG repeat size alleles was observed in both cell types from both twins, indicating the presence of extreme somatic instability and inter- and intra-tissue somatic mosaicism (**Figure 1B**). The *FMR1* mRNA expression levels in lymphocytes were ∼6-fold higher than age-matched control values, whereas the percentage of *FMR1* promoter methylation was approximately 3 and 2% in lymphocytes and 8 and 3% in fibroblast cell lines in twin 1 and in twin 2, respectively (**Table 2**), with undetectable promoter methylation in age-matched controls (Tassone et al., 2000). The mother carried *FMR1* alleles of identical CGG length (43, 78 CGG repeat) in both peripheral blood and fibroblasts, with an X-activation ratio (XAR; defined as the proportion of cells that carry the normal *FMR1* allele on the active X chromosome) of 0.15 and 0.17 respectively. *FMR1* expression levels were ∼2-fold higher than in control lymphocytes (**Table 2**).

**Table 2:** Triplet expansion, gene expression and methylation in FMR1 gene in samples from premutation carriers^∗^.

Outcome	Tissue	Controls	Twin 1	Twin 2	Mother
CGG repeats in each allele	Lymphocytes^∗^	28 ± 2 *(a)*	Premutation smear^∗∗^	Premutation smear^∗∗∗^	43, 78
	Fibroblasts	30 ± 1 *(b)*	Premutation smear^∗∗^	Premutation smear^∗∗∗^	43, 78
*FMR1* gene expression (fold change)^¶^	Lymphocytes^∗^	1 *(a)*	5.3	4.2	2.3
	Fibroblasts	1 *(c)*	1.3	−1.3	1.1
Methylation on *FMR1* promoter	Lymphocytes^∗^	<1% *(d)*	3%	<2%	XAR = 0.15
Q. 0.	Fibroblasts	NA	8%	3%	XAR = 0.17

(a) Control values (n = 12) have been published in Napoli et al. (2016b). (b)Control values (n = 12) have been published in Napoli et al. (2016a). (c) Control values were obtained from either male children (n = 5) with an average age of 8.8 ± 0.8 year (mean ± SEM) or adult females (n = 5) with an average age of 39.8 ± 2.5 year (mean ± SEM). (d) Taken from Tassone et al. (2000). ^¶^ Fold change referred to age- and sex-matched controls. ^∗^Analyses were performed in August 2013. ^∗∗^CGG = series of alleles throughout the premutation range with main alleles at 157 and 180 (see **Figure 1B**). ^∗∗∗^CGG = series of alleles throughout the premutation range with main alleles at 138 and 162 CGG (see **Figure 1B**). NA, not available. XAR, X-activation ratio.

In fibroblasts, the *FMR1* mRNA expression levels in the twins and their mother were not different from sex- and age-matched NC controls. The FMRP expression, as evaluated by Western blots, resulted in a main band at ∼80 kD whose levels were below the 95% CI. The same pattern was observed for the mother, albeit at a lower extent (**Figure 1C**).

### FMR1 Premutation Alters Mitochondrial Function and UPR^mt^

Average mitochondrial outcomes from the twins’ lymphocytes assessed at 3 different time points (**Supplementary Figure 2**) were compared to age- and sex-matched NC (**Table 3**). The selection of controls was based on the fact that *FMR1* is an X-linked gene and OXPHOS and other mitochondrial enzymes change with age (Rooyackers et al., 1996; Capkova et al., 2002). The most relevant changes in mitochondrial outcomes for both twins were the following: (a) decreased mitochondrial capacity to produce ATP fueled by both NAD- and FAD-linked substrates; (b) lower mitochondrial mass (citrate synthase activity), deficits in Complex IV and lower mtDNA copy number *per* cell, and (c) increased ROS/proton leak. The deficits in ATP production were (in most cases) proportional to those of mitochondrial mass (**Table 3**) indicating imbalances between mitochondria biogenesis (including mtDNA replication) and clearance, more than deficits at specific Complexes or segments of the electron transport chain. When mitochondrial outcomes tested in lymphocytes were compared between the twins, the bioenergetics from twin 2 resulted to be 39% lower than those of twin 1 (mitochondrial outcomes from twin 2 = 0.61 × mitochondrial outcomes from twin 1 + 0.16; *R*^2^ = 0.462; *p* < 0.0001; **Supplementary Figure 2**).

**FIGURE 2:**
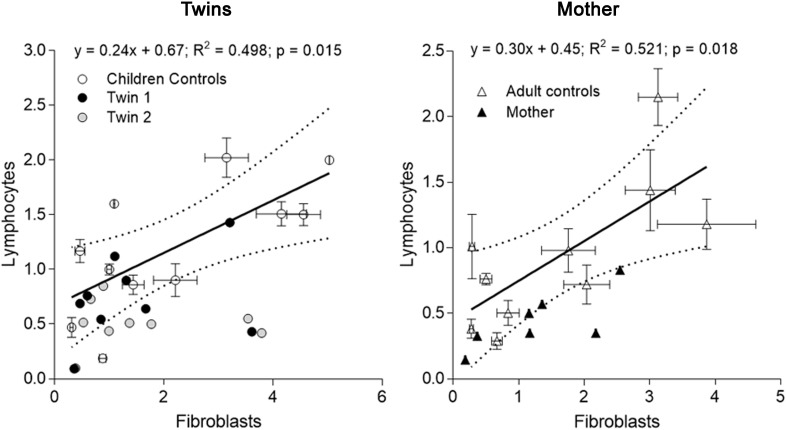
Correlation of mitochondrial outcomes between lymphocytes and fibroblasts from each carrier. Outcomes evaluated in lymphocytes (averaged from outcomes assessed at three different time points) of each twin were plotted vs. the same outcomes from fibroblasts (evaluated on 01/27/2014) (**Tables 3**, **4**). The same analysis was performed with the data from the mother. The linear regression (solid line) and the 95% CI (dotted lines) were built with control values. Outcomes outside of the 95% CI were FAD-dependent oxygen uptake, CCO, CCO/CS for twin 1; NAD- and FAD-dependent oxygen uptake, CCO, mtDNA copy number and CCO/CS for twin 2. Outcomes outside of the 95% CI evaluated with adult control values were for the mother: NAD-dependent oxygen uptake and CCO.

**Table 3:** Mitochondrial outcomes in lymphocytes from premutation twins and their parents.

Outcome	Children controls^¶^ (*n* = 10)	Twin 1	Twin 2	Adult controls (*n* = 7)	Mother	Father
**ATP-driven oxygen uptake Substrate**						
Malate-glutamate^∗^	1.6 ± 0.1 [1.4–1.8]	**0.9 ± 0.5**	**0.5 ± 0.1**	1.2 ± 0.2 [0.7–1.7]	**0.35 ± 0.04**	**0.3 ± 0.2**
Succinate^∗^	1.6 ± 0.2 [1.1–2.1]	**0.6 ± 0.2**	**0.4 ± 0.1**	1.4 ± 0.2 [0.9–1.9]	**0.35 ± 0.03**	**0.74 ± 0.07**
**Markers of mitochondrial inner membrane and matrix**						
Complex IV activity ^∗^	2.1 ± 0.4 [1.2–3.0]	**0.7 ± 0.3**	**0.8 ± 0.3**	2.2 ± 0.4 [1.2–3.2]	**0.8 ± 0.4**	1.4 ± 0.6
Citrate synthase activity^∗^	15 ± 2 [10–20]	**7.3 ± 1.4**	**4.8 ± 0.8**	7.6 ± 0.4 [6.6–8.6]	**1.45 ± 0.09**	**1.7 ± 0.3**
CCO/CS	0.14 ± 0.02 [0.09-0.19]	0.10 ± 0.05	0.17 ± 0.06	0.29 ± 0.06 [0.14–0.44]	0.6 ± 0.3	0.9 ± 0.4
**Others**						
RCRu	3.2 ± 0.3 [2.5–3.9]	3.0 ± 2.0	4.8 ± 3.0	8 ± 2 [3–13]	**1.1 ± 0.2**	6.9 ± 0.7
SRC (%)	239 ± 44 [139–339]	145 ± 26	275 ± 249	309 ± 48 [191–426]	350 ± 40	208 ± 20
ROS/Proton leak (%)	47 ± 5 [36–58]	**60 ± 24**	**70 ± 15**	37 ± 7 [20–54]	**325 ± 35**	30 ± 3
mtDNA copy number *per* cell	1692 ± 242^±^ [1089–2293]	**579 ± 49**	**508 ± 50**	ND	ND	ND
mtDNA deletions	1.00 ± 0.05^±^ [0.87–1.13]	0.89 ± 0.04	0.85 ± 0.14	ND	ND	ND

^∗^Expressed as [nmol × (min × 10^6^ cells)^−1^]. RCRu, Respiratory Control Ratio under uncoupling conditions; SRC, Spare Respiratory Capacity. SRC expressed as the percentage of FCCP-induced oxygen uptake rate over the baseline one. ROS/proton leak as the percentage of oligomycin-induced oxygen uptake rate over the baseline one. ^¶^ Controls values for OXPHOS and enzymatic activities were taken from (Giulivi et al., 2010) and are consistent with the historic pediatric values obtained in our laboratory. ^±^Control values of mtDNA copy number and deletions were obtained from control male children (n = 6) with an average age of 9.6 ± 0.7 year. Data are reported as mean ± SEM for controls whereas twins’ data are expressed as mean ± SD for premutation carriers (average values obtained at three different dates, i.e., 08/15/2013, 01/27/2014 and 03/24/2016). Bolded values are below the 95% CI limit, except ROS/proton leak which is above. Bold and underlined, values ≤ 30% of control average. ND = not determined due to the limited amount of biological material available.

When the outcomes from the twins were compared to their parents’ lymphocytes—obtained during the last visit—the mother’s outcomes were similar to those from the twins (i.e., decreased ATP production mainly attributed to lower mitochondrial mass, lower Complex IV activity, and decreased coupling with increased ROS/proton leak; **Table 3**), albeit to a lower extent. The father’s lymphocytes showed decreased ATP production mainly with NAD-linked substrates, also proportional to the decrease in mitochondrial mass (citrate synthase activity) compared to age-matched controls (**Table 3**). All other outcomes in the father’s lymphocytes were within control values (**Table 3**).

Milder changes in the bioenergetics of fibroblasts of the carriers were noted compared to those of lymphocytes. Fibroblasts from both twins showed a modest impairment in ATP production fueled by NAD-linked substrates, increased uncoupling, higher ROS/proton leak and lower Complex IV activity along with increased mtDNA deletions in twin 2’s cells (**Table 4**). It is possible that the increased ROS/proton leak observed in both twins results in ROS-mediated damage to Complex I, leading to lower NAD-linked ATP production (**Table 4**). Differently from lymphocytes, when outcomes from fibroblasts (**Table 4**) were compared between brothers, both seemed equally affected (mitochondrial outcomes from twin 2 = 1.12 × mitochondrial outcomes from twin 1 - 0.08; *r*^2^ = 0.908; *p* < 0.0001; **Supplementary Figure 2**).

**Table 4:** Mitochondrial outcomes in cultured fibroblasts from premutation twins and their mother.

Outcome	Children controls (*n* = 5)	Twin 1	Twin 2	Adult controls (*n* = 5)	Mother
**ATP-driven oxygen uptake**					
Substrate					
Malate-glutamate^∗^	4.6 ± 0.3 [3.8–5.4]	**3.21 ± 0.01**	**3.54 ± 0.06**	3.9 ± 0.8 [1.7–6.1]	2.2 ± 0.1
Succinate^∗^	4.2 ± 0.9 [1.9–6.5]	3.61 ± 0.06	3.8 ± 0.5	3.0 ± 0.4 [1.9–4.1]	**1.2 ± 0.1**
**Markers of mitochondrial inner membrane and matrix**					
Complex IV activity^∗^	3.2 ± 0.4 [2.1–4.2]	**1.7 ± 0.4**	**1.8 ± 0.3**	3.1 ± 0.4 [2.0–4.2]	2.6 ± 0.2
Citrate synthase activity^∗^	5 ± 1 [2.4–7.6]	4.7 ± 0.3	5.2 ± 0.3	5.0 ± 0.9 [2.5–7.5]	**1.9 ± 0.9**
CCO/CS	0.6 ± 0.1 [0.34–0.86]	0.36 ± 0.09	0.38 ± 0.07	0.67 ± 0.08 [0.45–0.89]	1.4 ± 0.7
**Others**					
RCRu	5.03 ± 0.50 [3.75–6.32]	**2.4 ± 0.9**	**3 ± 1**	4.7 ± 0.4 [3.6–5.8]	3.71 ± 0.03
SRC (%)	125 ± 8 [104–146]	124 ± 41	160 ± 30	127 ± 2 [121–133]	138 ± 10
ROS/Proton leak (%)	31 ± 3 [23–39]	**60 ± 40**	**66 ± 24**	28 ± 3 [20–36]	**37 ± 3**
mtDNA copy number *per* cell	1085 ± 120 [776–1394]	849 ± 10	996 ± 80	918 ± 95 [654–1182]	**1614 ± 17**
mtDNA deletions	1.00 ± 0.04 [0.93–1.07]	1.1 ± 0.1	**0.89 ± 0.04**	1.0 ± 0.2 [0.8–1.2]	1.1 ± 0.1

Experimental details were described under Methods. ^∗^Activities expressed as [nmol oxygen consumed × (min × 10^6^ cells)^−1^]. Data are shown as mean ± SEM for controls and as mean ± SD for premutation carriers. Between brackets, 95% CI built with control values obtained with fibroblasts from either male children with an average age of 8.8 ± 0.8 year (mean ± SEM) or adult females with an average age of 39.8 ± 2.5 year (mean ± SEM). Bolded values are below the 95% CI limit, except ROS/proton leak and mtDNA copy number which are above.

Fibroblasts from the mother showed similar decreases in both FAD-linked oxygen uptake and citrate synthase activity but compensated by an increased ATP production at the level of Complex I (i.e., NAD-linked oxygen uptake normalized by citrate synthase activity). A marginal increase in ROS/proton leak and the lack of changes in coupling are indicative of a better clearance of damaged mitochondria. The higher mtDNA copy number *per* cell indicates an underlying increased oxidative stress which triggers an increased replication of naive (undamaged) mtDNA template (**Table 4**).

When mitochondrial outcomes from lymphocytes were correlated to those obtained with their fibroblasts’, several outcomes, especially from twin 2, fell outside the 95% CI (5 vs. 3 for twin 1; **Figure 2**). In the mother, values for most outcomes from both cell types were within the 95% CI (**Figure 2**). In agreement with the substantial residual bioenergetics’ capacity of fibroblasts relative to other systems [even in cases of pathogenic mtDNA mutations (Thorburn and Smeitink, 2001)], mitochondrial parameters in lymphocytes from NC were 24–30% of fibroblasts from NC, indicative of a different cell-specific OXPHOS capacity.

Notably, the stoichiometric balance between components of OXPHOS complexes was disrupted in the carriers’ fibroblasts. In the case of the twins, these altered ratios seemed ascribed to lower Complex IV activity, whereas in the case of the mother to lower Complex II activity (within the succinate-dependent oxygen uptake; **Table 4**). Altered ratios of OXPHOS Complexes triggers the UPR^mt^ which entails the expression of mitochondrial chaperones to assemble subunits into multi-subunit functional complexes (Jovaisaite et al., 2014). Consistent with these findings, the majority (67%) of mitochondrial proteins, representative of four mitochondrial sub-compartments (outer membrane, intermembrane space, inner membrane and matrix), were higher in twin 2’s cells; conversely, 44% and 22% showed higher abundance in twin 1’s and mother’s cells, respectively (**Supplementary Figure 3**). These results suggested an accumulation of misfolded, inactive mitochondrial proteins in twin 2’s cells, likely through a disrupted UPR^mt^, and consistent with the accumulation of damaged mtDNA (**Table 4**).

**FIGURE 3:**
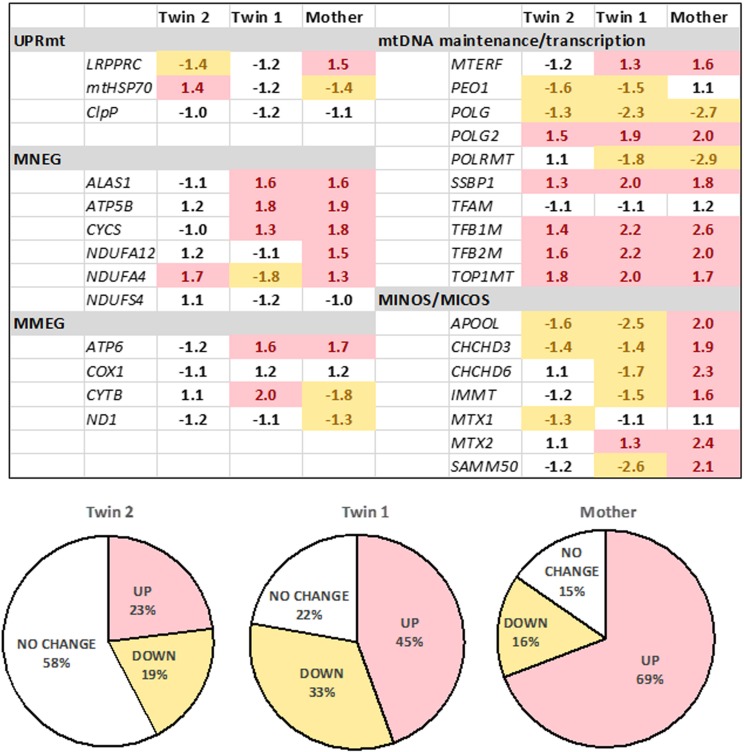
Expression of mitochondrial genes in fibroblasts from the twins and their mother. The gene expression was evaluated by qRT-PCR utilizing dual-labeled probes. Gene expression is reported as log 2 fold-change relative to age- and sex-matched control values (*n* = 5 for each group) and was calculated as described in the Materials and Methods and **Supplementary Methods**. Pie charts show percentages of upregulated, downregulated, or unchanged gene expression in the twins and the mother. UPR^mt^, mitochondrial unfolded protein response; mitochondrial products encoded by the nuclear genome (MNEG, mitochondrial nuclear-encoded genes) or by the mitochondrial DNA genome (MMEG, mitochondrial mtDNA-encoded genes); mtDNA, mitochondrial DNA; MICOS, mitochondrial contact site and cristae organizing system complex (also known as MINOS or MitOS Complex).

To test the status of UPR^mt^, we evaluated the gene expression of three key genes (i) the leucine-rich pentatricopeptide repeat motif-containing protein [*LRPPRC*; also known as *LRP130*] also involved in autophagy/mitophagy and Complex IV activity; (ii) the mitochondrial chaperone *HSP70* and (iii) the mitochondrial protease *CLPP*. These experiments were complemented by assessing the expression of selected genes involved in mtDNA maintenance, OXPHOS subunits and assembly factors, as well as the mitochondrial disulfide relay system. The gene expression of *LRPRRC* and *HSP70* was consistent with the activation of UPR^mt^ in twin 2’s cells, whereas no changes (twin 1’s cells) or the opposite trend was observed in the mother’s cells. Analysis of the remaining 27 genes (**Figure 3**) showed no changes in the expression of the majority of genes in twin 2 (58% within NC values), whereas in twin 1 and his mother, the majority was up-regulated (45% and 69%, respectively).

Taken together, the gene expression of *LRPRRC* and *HSP70*, the accumulation of mitochondrial proteins (**Supplementary Figure 3**) and damaged mtDNA (**Table 4**) were consistent with a defective activation of the UPR^mt^ avoiding mitophagy in an attempt to preserve some OXPHOS activity (Lin et al., 2016). In the mother’s and twin’s 1 cells, while no activation of UPR^mt^ was observed, the up-regulation of most genes tested suggested an attempt to balance mitochondrial biogenesis, mitochondrial proteostasis and mitophagy (Jin and Youle, 2013).

### Disrupted Mitochondria Morphology, Mass and Network in Fibroblasts From PM Carriers

**FIGURE 4:**
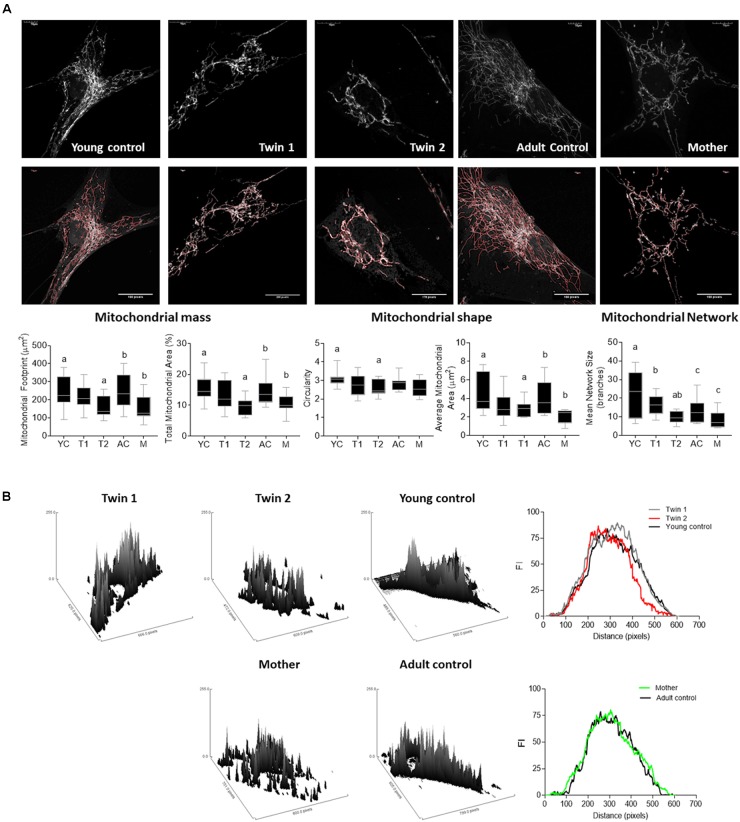
Mitochondrial mass, morphology and distribution in fibroblasts from twins, their mother and relative controls. **(A)** (Top) Mitochondrial staining was carried out with MitoTracker Red CMXRos (as described in the Materials and Methods) and shown in gray scale. (Middle) The outline of the mitochondrial network obtained with the MiNA macro for Fiji is shown in red. Bottom. Parameters assessed by this plugin include mitochondrial footprint, total and average mitochondrial area, circularity index, and network size. YC, young controls; AC, adult controls; T1, Twin 1; T2, Twin 2; M, Mother. **(B)** Representative plots and histogram outlines showing mitochondrial distribution in fibroblasts from twins, their mother and respective controls. Images were obtained with the surface plot feature and analyzed with the plot profile tools in Fiji. Histogram outlines were obtained averaging data from at least 10 images *per* cell line. On the *x* axis is the distribution of the fluorescence intensities (FI). Values that are further from the center of the axis represent mitochondrial with less perinuclear localization.

To test the steady-state of dysfunctional mitochondria, fibroblasts were stained with MitoTracker Red CMXRos (dye that requires polarized mitochondria for its accumulation) and visualized by confocal microscopy. Acquired images were subsequently analyzed to allow the quantification of mitochondrial mass, morphological features and network integrity (Dagda et al., 2009; Valente et al., 2017; **Figure 4A**). A decreased mass of polarized mitochondria was observed in fibroblasts from twin 2 and the mother compared to their respective age- and sex-matched NC. This was supported by the decreased mitochondrial footprint (defined as the total mitochondrial area after being separated from the background; **Figure 4A**) as well as the reduced mitochondrial content *per* cell (percentage of cell area occupied by mitochondria). A shift from the mitochondrial tubular shape (characteristic of control cells) to smaller, more punctuated structures was observed in twin 2, indicating either a prevalence of mitochondrial fission over fusion. A disrupted mitochondrial network (as judged by the mean number of branches *per* network; **Figure 4A**) was observed in fibroblasts from all carriers compared to controls. Surface plot analysis of the cellular mitochondria distribution indicated a distinct, yet modest perinuclear mitochondria distribution only in twin 2’s cells compared to both age-matched NC and twin 1 (**Figure 4B**).

Taken together, the increased perikarya clustering observed in cells from twin 2 (**Figure 4B**), the functional and dynamic deficits observed in the fibroblasts of the carriers (**Figure 4A**), increased uncoupling in twins (**Table 4**), increased mass of depolarized mitochondria in twin 2 and mother (**Figure 4A**), were all findings consistent with not only altered mitochondrial homeostasis but also their distribution and cytoskeleton reorganization (Chen and Chan, 2006).

### Mitochondrial ROS-Dependent HIF-1a Stabilization Underlies the PM-Induced Bioenergetics Deficits

**FIGURE 5:**
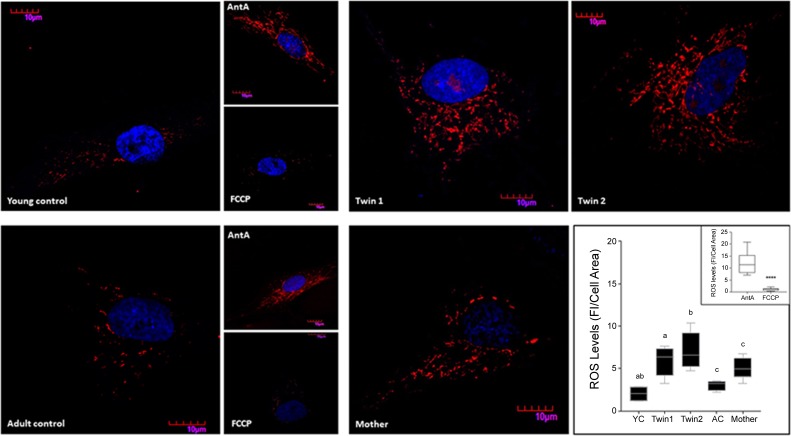
Mitochondrial ROS production in fibroblasts from twins, mother and controls. Representative images of ROS levels in fibroblasts from twins, their mother, and young (YC) and adult (AC) controls were obtained upon labeling of the cells with 5 μM MitoSOX as described in the Section “Materials and Methods.” Fluorescence intensity (FI) was analyzed with Fiji, normalized by cell area and shown as the median with intervals. Statistical analysis was performed with the one-way ANOVA followed by Kruskal–Wallis *post hoc* test for twins and respective controls and with Mann–Whitney *t*-test between mother and age-matched controls. *P*-values are as follows: a = 0.0255, b = 0.0047, c = 0.0317. Young and adult control cells pre-treated with antimycin A and FCCP were used as positive and negative controls respectively. Fluorescence intensities of cells exposed to antimycin A and FCCP normalized by cell area are shown in the inset. A Mann–Whitney *t*-test was performed between the two groups. ^∗∗∗∗^*p* < 0.0001.

As increased mitochondrial ROS production is often associated with the accumulation of defective mitochondria, we tested for mitochondrial superoxide anion production by utilizing MitoSOX staining. The specificity of the staining for mitochondrial ROS was confirmed by using NC fibroblasts which showed negligible staining in the presence of an uncoupler (FCCP, which decreases ROS production) and an intense and punctuated staining obtained with antimycin A (increases ROS production at Complex III; **Figure 5**). A significant higher mitochondrial superoxide anion production was observed in the fibroblasts from the carriers (**Figure 5**), resulting in 2.9-, 3.5-, and 1.7-fold of sex- and age-matched NC for twin 1, twin 2 and mother, respectively. These data are consistent with the observed increases in oligomycin-resistant oxygen uptake (**Table 4**).

**FIGURE 6:**
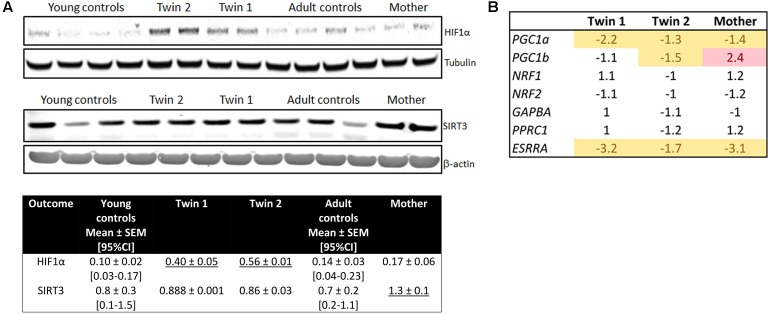
Mitochondrial biogenesis and levels of HIF-1α and SIRT3 in fibroblasts from twins, their mother and controls. **(A)** Representative Western blot images and densitometry of HIF-1α levels in fibroblasts from controls, twins and their mother. Data are shown as mean ± SEM for controls. Mean ± SD was used for twins and their mother’s values as an index of intra-experimental variability. Reported are also the limits of the 95% CI obtained with age- and sex-matched controls (between brackets). Underlined values are higher than the upper limit of the 95% CI. **(B)** Expression of genes (by qRT-PCR utilizing dual-labeled probes) involved in mitochondrial biogenesis.

As increased mitochondrial ROS production can stabilize and prevent the degradation of HIF-1*α* (Bardos and Ashcroft, 2005), thereby decreasing mitochondria proliferation, mitochondrial mass and metabolism (Semenza, 2011), we tested the protein expression of HIF-1*α* as well as that of its downstream target, the hypoxia up-regulated mitochondrial movement regulator protein (HUMMR; Li et al., 2009), and several others genes involved in mitochondrial biogenesis. Despite that all PM carriers’ cells showed higher mitochondrial levels of ROS/proton leak (**Table 4**) and mitochondrial ROS (**Figure 5**), the protein levels of HIF-1α and HUMMR were significantly increased only in the twins’ cells relative to age-matched NC (**Figure 6A** and **Supplementary Figure 3**). Twin 2’s values were 1.4-fold of his brother’s, mirroring the higher mitochondrial ROS production (1.2-fold). Contrary to the expectation that HIF-1α stabilization would lead to a switch from OXPHOS to glycolysis, and as such an increase in the [lactate]-to-[pyruvate] ratio (L/P), a higher L/P was only observed in twin 2’s cells (L/P = 24; 95% CI = 16.3–19.6; *n* = 6). This higher L/P ratio in twin 2 was accompanied by a down-regulation of most genes involved in mitochondria biogenesis (including *PGC-1*α and *PGC-1β*; **Figure 6B**). The protein expression of the mitochondrial NAD^+^-dependent sirtuin deacetylase SIRT3 [a target of PGC-1α, suppressor of mitochondrial ROS and a factor in mitochondrial biogenesis (Kong et al., 2010)] was higher only in the mother’s cells than age-matched NC (**Figure 6A**).

## Discussion

Mitochondrial dysfunction has emerged as a common mechanism underlying many neurological (Ross-Inta et al., 2010) and neurodevelopmental disorders (Giulivi et al., 2010), characterized by lower ATP production, increased mitochondrial ROS, or metabolite-inhibition of critical steps within mitochondrial pathways (Johri and Beal, 2012). MD has been previously reported in fibroblasts from young adults and older PM carriers with a direct correlation between severity of MD and the occurrence of clinical symptoms (Ross-Inta et al., 2010; Napoli et al., 2011). For the first time we hereby report an in-depth characterization of MD in 8 years old PM MZ twins presenting severe emotional and psychiatric deficits, not accompanied by intellectual and/or cognitive issues. At a molecular level both twins were characterized by a broad size range of CGG repeats at the upper limit, accompanied by lower FMRP levels than age-matched NC values. This outcome could be explained by deficits in the translational efficiency, as previously reported for other carriers, particularly when carrying *FMR1* alleles in the upper PM range (Primerano et al., 2002).

In search for a mechanism underlying the observed clinical phenotypes, and considering the critical reliance of brain on mitochondria-derived ATP, we characterized in detail the status of mitochondrial function, morphology and distribution, oxidative stress, and related signal transduction pathways in their dermal fibroblasts and lymphocytes, both proven to be suitable *in vitro* models for the study of CNS (Ross-Inta et al., 2010; Napoli et al., 2016b). Although it could be argued that testing for both cell types might be redundant, the collective information is complementary based on differences in (i) germ layer lineage (fibroblasts, as neurons, are ectodermal in origin, whereas immune cells are derived from the mesoderm); (ii) environmental milieu (carrier’s environment in lymphocytes vs. fibroblasts grown under optimal conditions and removed several generations from the carrier); (iii) and proliferative status (terminally differentiated cells vs. proliferative ones in which the redox status of the latter is more oxidative to match the higher ATP demands needed for rapid growth).

In the twins’ lymphocytes, the deficits in OXPHOS were (in most cases) proportional to those of mitochondrial mass, indicating an imbalance between mitochondria biogenesis (including mtDNA replication) and clearance. This imbalance was reflected as an accumulation of damaged mitochondria with higher ROS/proton leak and lower Complex IV activity. In this regard, it is likely that enhanced mitochondrial ROS-mediated inhibition of the prolyl hydroxylases-dependent degradation of HIF-1*α* (Bardos and Ashcroft, 2005) stabilized this factor under normoxic conditions, ensuing in decreased mitochondria proliferation, mass and metabolism (Semenza, 2011), more pronounced in twin 2’s cells.

The mitochondrial abnormalities recorded in twin 2’ fibroblasts indicated a decrease in the repair mechanism of damaged mitochondrial proteins and/or clearance of damaged mitochondria. These events were accompanied by lower *PGC-1α* and *PGC-1β* gene expression, and HIF-1*α* stabilization with a defective UPR^mt^ activation. The bioenergetics’ defects observed in twin 2’s fibroblasts were considerable, for some of them crossed the threshold for energy deficiency, consistent with the repression of both transcriptional *PGC* co-activators and the lack of compensation by the increased glycolysis. This is supported by a ratio of mitochondrial-to-glycolysis ATP of 0.6, significantly lower than that of NC (1.0), and near the energy threshold for brain OXPHOS (≤50% of Complex I activity (Rossignol et al., 2003)), segregating with the worse phenotype. While the lower *LRPPRC* gene expression in twin 2’ cells should have triggered basal autophagy (Zhang et al., 2017) and mitophagy (Zou et al., 2013), the accumulation of damaged/dysfunctional mitochondria (i.e., increased depolarized mass) could be explained by considering that LRPPRC activity requires its association with cytoskeleton elements participating in vesicular distribution of both membrane and nuclear organelles (Xie et al., 2011), processes involving the formation of vesicles and their trafficking through the cytoskeleton network (such as mitophagy and autophagy) which could be affected or halted when FMRP is deficient. The lower gene expression of *LRPPRC* might also contribute to the significant OXPHOS deficits and lower Complex IV activity, considering that LRPPRC is a component of the PGC-1*α* complex linked to cellular energy homeostasis (Zhang et al., 2017) and that pathogenic *LRPPRC* mutations result in impaired Complex IV assembly and activity (Xu et al., 2004).

The bioenergetics of fibroblasts from twin 1 showed an intermediate situation between his brother and mother. The control levels of both PGC-1*β* and SIRT3 seemed to partly counteract the downstream effects triggered by the mitochondrial ROS-dependent stabilization of HIF-1α resulting in no increases in the glycolytic flux (control L/P ratio) with no significant accumulation of damaged mitochondria, but still milder OXPHOS deficits.

In the case of the mother, fewer outcomes seemed affected, likely *via* activation of some compensatory mechanisms, thereby preventing profound energy deficits. The imbalanced stoichiometry between components of OXPHOS complexes triggered the mitochondrial-to-nuclear signaling to increase UPR^mt^ response to maintain protein quality and mitochondrial function (Pellegrino et al., 2013). This is supported by the relatively milder mitochondrial ROS levels in the mother’s cells (not enough to activate HIF-1α) and the higher content of more depolarized mitochondria (which could be interpreted in the context of all results as an increase in OXPHOS). Notably, the higher expression of *PGC-1β* in the mother’s cells (but not that of *PGC-1α*) may suggest the conservation of mitochondrial fatty acid β-oxidation (Kamei et al., 2003), process that fuels less pro-inflammatory stress responses (Pellegrino et al., 2014). The increased gene expression of *LRPPRC*, higher mtDNA copy number, normal L/P, and up-regulation of SIRT3, *PGC-1*β, and UPR^mt^ seem enough to sustain an adequate OXPHOS activity in the mother’s cells but not to preserve the mitochondrial network, process that relays on the integration with cytoskeletal proteins (Xie et al., 2011).

A relevant question is which biochemical outcomes associate with any of the clinical diagnoses received by the twins and their mother. One possible scenario is that mitochondrial deficits shared by both twins may underlie the pathogenic mechanisms leading to the neurological symptoms present in both children (depression, mood instability, ADHD), whereas others (e.g., a more severe OXPHOS deficit, higher mitochondrial ROS production, repression of PGC-1*α*/β) may associate with the bipolar disorder, maniac behavior, and chronic fatigue diagnosed solely in twin 2. Hence, these differences may be rooted on additional environmental stress imposed by the chronic inflammation triggered by Lyme disease in twin 2, given that the degree of the pro-inflammatory immune responses is fueled by the balance between glucose vs. fatty acid oxidation (Arnoult et al., 2011). Similarly, the lower lymphocytic citrate synthase activity observed in both parents compared to age-matched NC, may point to shared environmental stress factors, whereas the outcomes found deficient in the mother only (vs. her spouse) may be attributed to her carrier status. Although a MD was observed in the mother’s cells at a lower extent than the twins, her unfavorable XAR in both lymphocytes and fibroblasts and the high *FMR1* mRNA levels in peripheral blood could play a role in her FXPOI condition, as it has been widely reported in the PM (Sherman, 2000). A key implication of these findings is that the different degree of severity in the twins vs. their mother may differ not only because of their contrasting genetic background (heterozygous with 78 CGG vs. hemizygous with a series of alleles spanning the upper range of the PM range), but also because of their sex-steroid environment, particularly the beneficial effects of estrogen on mitochondrial biogenesis and quality control factors (Hara et al., 2014).

Given the role of mitochondria in providing ATP to aerobic tissues such as brain, as well as their involvement in neurotransmitter metabolism (Laughlin et al., 1998), it is tempting to propose that the mitochondrial defects identified in this study are either causals or contributors of the emotional/developmental problems in a PM background, exacerbated by other factors (Lyme disease, sex hormones, XAR). Although other contributing factors cannot be ruled out, the observed changes in the twins’ cells may originally stem from a FMRP-dependent cellular dysregulation (not necessarily related to *FMR1* gene expression). FMRP is a polyribosome-associated neuronal RNA-binding protein, and relatively lower protein levels might ensue in cellular stress with the loss of translational brakes on the synthesis of critical proteins and factors required to sustain synaptic (e.g., synaptic long-term potentiation, glutamate receptor signaling, CREB signaling in neurons) and other intracellular signaling pathways (Darnell et al., 2011), including those possibly involved in bioenergetics as well as trafficking. Our study identified mitochondrial ROS- HIF-1α crosstalk as one of the central pathways in the MD, suggesting the therapeutic potential of targeting HIF-1α by small-molecule inhibitors or modulating mitochondrial ROS production.

### Study Limitations

This case report includes a detailed clinical and molecular description of two MZ twins showing remarkable inter- and intra-tissue somatic mosaicism with relatively long CGG repeats and no cognitive/intellectual issues but profound emotional/behavioral ones. Therefore, the conclusions should not be extended to the entire PM pediatric population. Furthermore, at the time of the assessments both twins were receiving several medications for the management of behavioral traits. Although none of these medications appear to impact directly or significantly mitochondrial function, due to the few and somewhat contradictory reports available [methylphenidate (Fagundes et al., 2010), sertraline (Chen et al., 2014), and aripiprazole (Ota et al., 2012)], an effect of these drugs on the lymphocytic (but not fibroblasts) outcomes tested in this study cannot be entirely ruled out. If indeed these medications had a detrimental effect on mitochondrial function, then cautionary advice should be exercised when prescribing them to subjects with suspected mitochondrial disorders.

## Author Contributions

EN carried out all polarographic measurements, confocal imaging, some immunoblotting experiments, statistical analyses, contributed to the writing of the manuscript, and revised and approved the final version as submitted. AS provided psychiatric and psychological assessment of children, revised the manuscript, and approved the final manuscript as submitted. RH carried out clinical assessment of these children and wrote clinical findings, revised the manuscript, and approved the final manuscript as submitted. GS carried out enzymatic measurements, some immunoblotting experiments, and approved the final manuscript as submitted. SW performed all experiments related to mtDNA, gene expression and approved the final manuscript as submitted. FT provided molecular data for **Table 2** and **Figure 1**, revised the manuscript, and approved the final manuscript as submitted. CG conceptualized and designed the study, wrote most of the manuscript, and approved the final manuscript as submitted.

## Conflict of Interest Statement

RH has received funding from Novartis, Roche/Genentech, Marinus, and Neuren for treatment trials in fragile X syndrome, autism and Down syndrome. She has also consulted with Zynerba and Fulcrum regarding treatment for fragile X syndrome. The remaining authors declare that the research was conducted in the absence of any commercial or financial relationships that could be construed as a potential conflict of interest.

## Abbreviations

ADHD: attention-deficit/hyperactivity disorder
ASD: autism spectrum disorders
*FMR1*: fragile X mental retardation 1
FMRP: fragile X mental retardation protein
FXS: fragile X syndrome
FXPOI: fragile X-associated primary ovarian insufficiency
FXTAS: fragile X-associated tremor and ataxia syndrome
HIF-1α: hypoxia-inducible factor 1-alpha
L/P: lactate-to-pyruvate ratio
MD: mitochondrial dysfunction
MZ: monozygotic
NC: non-carriers
OXPHOS: oxidative phosphorylation
PM: premutation
qRT-PCR: quantitative-real time PCR
ROS: reactive oxygen species
UPR^mt^: mitochondrial unfolded protein response
XAR: X-chromosome activation ratio
