# Supplementary material for: Impact of FMR1 Premutation on Neurobehavior and Bioenergetics in Young Monozygotic Twins
Source: Front Genet. 2018 Aug 27;9:338. doi: 10.3389/fgene.2018.00338 (PMC6119880; doi:10.3389/fgene.2018.00338)
Supplement: Supplementary file 1 [file Data_Sheet_1.PDF]

# Supplementary Information

## Impact of FMR1 premutation on neurobehavior and bioenergetics in young monozygotic twins

Eleonora Napoli[1], Andrea Schneider[2,3], Randi Hagerman[2,3], Gyu Song[1], Sarah Wong[1], Flora Tassone[2,4], and Cecilia Giulivi[1,2]

[1] Department of Molecular Biosciences, University of California Davis, School of Veterinary Medicine, Davis, CA

[2] UC Davis MIND Institute, UC Davis Health, Sacramento, CA

[3] Department of Pediatrics, School of Medicine, University of California Davis, Sacramento, CA

[4] Department of Biochemistry and Molecular Medicine, School of Medicine, University of California Davis, Sacramento, CA

**Address correspondence to**: Dr. Cecilia Giulivi, Department of Molecular Biosciences, University of California, 1089 Veterinary Medicine Dr., VetMed 3B #3009, Davis, CA 95616, cgiulivi@ucdavis.edu, 530-752-1038

**Supplementary detailed medical history of the subjects reported in the study-**Two 7 years old identical twins were recruited through the Fragile X Treatment and Research Center at the MIND Institute at UC Davis Medical Center. The UC Davis Institutional Review Board approved all aspects of the study and informed consent was obtained from the parents of the children. Consent to publish this case reports was also obtained from the parents.

Typical neurodeveloping and age-matched control children (aged 2-8 y old) were enrolled through the CHildhood Autism Risks from Genetics and Environment (CHARGE) study as described before (Giulivi et al., 2010). The twin pregnancy was normal and mother was induced at term and delivered by C-section. Both twins weighed 5 lb 13 oz. They developed normally in the newborn period. They were breastfed for the first six weeks and they thrived.

**Twin 1** sat at 6 months, crawled at 10 months, walked at 15 months, and said words at 2 years and phrases at about 3.5 years of age. His speech was cluttered and also tangential but he was very talkative. He began receiving speech and language therapy at about 3 years of age, and he was diagnosed with a phonological disorder. He has also received occupational therapy for sensory-motor deficits, and he has been in a private school in a different classroom than his brother. In the past, the behavior of Twin 1 has included chewing on his sleeves, ADHD symptoms and poor eye contact. He is tactilely defensive and quite hyperactive. He is hypersensitive to stimuli including noises, smells and tastes, and seems to be more sensitive to sensory inputs than his brother. He has been obsessed with food at times with intermittent hyperphagia. He was treated with stimulants for his ADHD symptoms beginning at age 6, initially with Vyvanse and then switched to Concerta, which seemed to work better for him at age 7. His treatment regimen has included long acting guanfacine for tantrums and sleeping difficulty and 50 mg a day sertraline for anxiety. He also takes melatonin for his sleeping difficulties. He has not had the same rage outbursts as his brother, but he can tantrum. He is interested in social interactions and friendly but he can be easily overwhelmed by social situations. He tends to be more intense and anxious compared to his brother. His medical history includes an inguinal hernia repair at 3.5 years, approximately five otitis media infections in the first 5 years of life, reactive airway disease intermittently treated with a bronchodilator inhaler and occasional use of oral steroids and more frequent use of nasal steroids.

On examination at age 7 his blood pressure was 88/56, head circumference 52.5 cm, height 118.1 cm (15$^{th}$ percentile) and weight is 18.96 kg (3.8$^{th}$ percentile). His speech was fast and occasionally cluttered. HEENT (head, eyes, ears, nose and throat) exam was normal and his ears were not prominent but his palate was high arched. His finger joints were mildly hyperextensible at the metacarpal phalangeal joint. He was able to tandem walk without difficulty, finger-to-nose touching was normal, deep tendon reflexes were 1+ in the upper extremities, 2+ at the knees and ankles and the rest of his neurological examination was normal. He was tanner stage I with normal sized testicles. Therapy was initiated with a psychologist to treat his anxiety, ADHD symptoms, and to teach him to self-calm in over-stimulating situations. His medication regimen has included 250 mg *N*-acetyl-cysteine (NAC) twice a day and a variety of antioxidants, including B vitamins and omega 3s along with medications outlined above. He subsequently developed depressive thinking, a poor self-image and paranoid ideation, so he was started on aripiprazole with significant improvement in his thinking and a calming of his behavior problems. On cognitive testing at age 8 with the WISC-IV (Weschler Intelligence Scale for Children-IV), his Verbal Comprehensive Index (VCI) was 136, Perceptual Reasoning Index (PRI) was 112, Visual Motor index (VMI) was 109, and Full-Scale IQ was 123, so he is gifted. His memory scores were in the mid average range (100-109). On the Autism Diagnostic Observation Schedule (ADOS) his score was 4 on repetitive

and restrictive behavior and his total score was 4, normal range, with no problems related to social affect. He was also given the Kiddie-Schedule for Affective Disorders and Schizophrenia (KSADS) and he met criteria in the past for major depressive disorder in partial remission. This was at age 5 when he was having some suicidal ideation. He met criteria for agoraphobia in the past in partial remission and specific phobia with animals. He also had an eating disorder with stuffing his mouth and he had significant ADHD symptoms.

**Twin 2** began sitting at 6 months, crawled at 9 months, walked at about 14 months, but he was delayed in language with first words said at about 2 years and phrases at about 3.5 years of age, when he initiated speech and language therapy. He had ongoing articulation problems along with pragmatic deficits. At age 7, he started receiving physical and occupational therapy twice a week. He was also in a private school with a small number of children and he did very well in the classroom. His behavior included some intermittent difficulties with eye contact. He was on the hyperactive side, but he enjoyed hugging people. He was "craving" of tactile input and he also liked to snuggle, but he was easy to anger, and he had a tendency to withdraw. At age 6, he had some violent imagery and vocalization, which escalated to tantrum behavior. They were rage episodes within a few months, so he was placed on 0.5 mg/day risperidone but his appetite dramatically increased. He was subsequently tried on sertraline at 25 mg and this was immediately helpful for his anger and depression, but one month later when it was increased to 50 mg a day, he became manic and was seen in the emergency room with a diagnosis of bipolar disorder. His sertraline was decreased to 25 mg and the risperidone was switched to 1 mg aripiprazole in the morning and he did much better. Although he had ADHD behavior, stimulants were activating for him although a low dose was tolerated, and long acting guanfacine has been somewhat helpful. He also took antioxidants including NAC and omega 3s. His behavior included some perseveration. His violent ideation and outbursts have improved but he become impulsive when upset. He was not shy with others, but he was somewhat disinterested in others, although he did not have autism. He had ongoing anxiety issues, along with moodiness. He had few, if any, friends and he felt somewhat disinterested in them. Sometimes, he could feel persecuted by others, but this was only intermittently felt. He raged at times if he did not get what he wanted to eat, but he had a very narrowed focus of what he was willing to eat. He also had very significant motor coordination problems and poor stamina, and this interfered with his participation in sports. His physician diagnosed him with an adjustment disorder, along with developmental motor coordination deficits and ADHD. His past medical history included five ear infections, a repair of a right inguinal hernia and an umbilical hernia, which resolved in the first few years of life. He has had a history of reactive airway disease and seasonal allergies. Most important is his history of Lyme disease at age 6 with erythema migrans under his arms, and treated with amoxicillin. He also had a fairly significant headache with the Lyme disease, but this eventually resolved. On examination, his blood pressure was 93/50, head circumference was 52.5 cm, height was 119.9 cm (19.6$^{th}$ percentile), and weight was 22.5 kg (34$^{th}$ percentile). He had intermittent poor eye contact during the examination, but he laughed and cooperated well. On the finger-to-nose touching, he became a little bit oppositional, but when the physician described that he would get lunch quickly, he subsequently sped up his responses. His finger joints were hyperextensible with MP extension to 90 degrees but his feet were not flat and his ear pinna were not prominent. The rest of his physical and neurological examination was normal for age. At age 7 he was given the KSADS which documented depressive disorder in partial remission, bipolar disorder, ADHD and evidence of mania current and past in addition to chronic fatigue. His cognitive testing on the WISC-IV demonstrated a VCI of 112, PRI of 82, Processing Speed Index (PSI) of 106 and Full-Scale IQ of

99.  From October 2013 to January 2014, he was taking 18 mg Concerta in AM, 5 mg in PM, 25 mg sertraline in AM, 2 mg aripiprazole twice a day and 300 mg NAC bid in addition to a multiple vitamin and omega 3s, similar to his brother.

**Mother** was a 38-y premutation carrier (CGG 43, 78), with no psychiatric or medical problems associated with the premutation. However, she describes herself as shy with poor eye contact as a child. After receiving the diagnosis of the twins, she experienced some anxiety and major depressive symptoms. At age 36, she met criteria for fragile X-associated primary ovarian insufficiency (FXPOI), reported some word retrieval problems and monthly migraines.

**Supplementary Methods**

**Evaluation of gene expression in fibroblasts and determination of the most stable housekeeping gene(s)** – The qRT-PCR was performed in a Mastercycler EP Realplex thermocycler (Eppendorf, Westbury, NY). Samples were pipetted in a 96-well plate with each reaction containing: 6 µl of TaqMan 2x Universal PCR Master Mix (Applied Biosystems), 0.2 µl of 20x primer-probe mix (Life Technologies), 0.8 µl nuclease-free water, and 5 µl of 200 ng total cDNA template. Amplification was performed using the following cycling parameters: 2 min at 50°C, 95°C for 10 min, followed by 40 cycles of 15 s at 95°C and 1 min of at 60°C. The mean cycle time was obtained by double derivatives (CalqPlex algorithm; Eppendorf, Westbury, NY) and designated as Ct. Each sample was analyzed in triplicates, and positive and negative controls were run on each plate. The gene expression for each target gene was determined by the comparative Ct method using the following equation: $2^{\Delta Ct}$, where $\Delta Ct = Ct_{Target}\text{-}Ct_{Housekeeper}$. The gene expression fold change (Carrier/non-carrier) was determined using the ΔΔCt method using the following equation: $2^\wedge(\Delta Ct_{Carrier}\text{-}\Delta Ct_{Non\text{-}carrier})$. Fold changes were calculated based on their age matched controls. Values were converted to positive or negative values to indicate up or down gene regulation, by log transforming the values X and then taken as: IF(X > 0, POWER (2,X), -1*POWER (2, -X) in Microsoft Excel. A cut-off value of ± 1.3-fold change was considered up or down regulated for a particular gene.

To determine the most stable housekeeping genes for normalization, geNorm and Normfinder were used. Underlying principles and formulas for the geNorm VBA applet (version 2002) have been previously described (Vandesompele et al., 2002) as well as the model and statistical framework for NormFinder (Andersen et al., 2004). GeNorm calculates the gene expression stability measure *M* for a reference gene as the average pairwise variation *V* for a particular gene with all other tested reference genes. Stepwise exclusion of the gene with the highest *M* value allows ranking of the tested genes according to their expression stability. The highest *M* values, which are the least stable, are eliminated until the best reference gene for normalization is found. Similarly, NormFinder ranks the set of candidate normalization genes according to their expression stability in a given sample set, but also shows the variation between sample subgroups of the sample set. Based on our comparison of geNorm and Normfinder, of the eight housekeeping genes tested, *XRCC5* was found to be the most stable gene whereas *ACTB* was the least (**Supplementary Figure 1**), thus *XRCC5* was used for the normalization. Gene expression stability for the other 6 housekeeping genes were ranked similarly, although not necessarily in the same order.

**Evaluation of mtDNA copy number and deletions-**These assays were performed on gDNA from lymphocytes by using qRT-PCR and essentially as described in detail in (Giulivi et al., 2010; Napoli et al., 2013b). The gene copy number of *ND1* was normalized by the single-copy nuclear gene, *PK* (pyruvate kinase). For mtDNA deletions, *ND4* and *CYTB* were normalized to *ND1*. Species-specific primers were selected using the Primer Express 3 software (Applied Biosystems). Primers for *PK* were: forward 5'-AGCCCAAATGGCCTTGAAG-3'; reverse 5'-AGAGACAGAATGCCAGTGAGCTT-3'; primers for *CYTB* were: forward 5'-CACGATTCTTTACCTTTCACTTCATC-3'; reverse 5'-TGATCCCGTTTCGTGCAAG-3'. The probes used were from Roche UPL library, Locked Nucleic Acids, short hydrolysis probes, labeled at the 5' end with fluorescein and at the 3' end with a dark quencher dye (#11 for *PK*, cat no: 04685105001; #10 for *CYTB*, cat no: 04685091001). *ND1* primers and probe were: forward 3485-3504, 5'-CCCTAAAACCCGCCACATCT-3'; reverse 3532-3553, 5'- GAGCGATGGTGAGAGCTAAGGT-3'; probe

3506-3529, 5’ FAM-CCATCACCCTCTACATCACCGCCC-BHQ-3’. *ND4* primers and probe were the following: forward 12087-12109, 5’-CCATTCTCCTCCTATCCCTCAAC-3’, reverse 12140-12170, 5’ -CACAATCTGATGTTTTGGTTAAACTATATTT-3’, probe 12111-12138, 5’FAM-CCGACATCATTACCGGTTTTCCTCTTG-BHQ-3’. The qRT-PCR was performed on a Mastercycler EP Realplex thermocycler (Eppendorf, Westbury, NY). The corresponding qRT-PCR efficiencies for each mitochondrial and nuclear gene amplification were calculated from experimental data according to the equation: $E = 10^{(-1/slope)} -1$ according to the Mastercycler EP manual. After establishing the linear response between Ct number and template amount (25, 12.5, 6.25, 3.13 and 1.56 ng total per reaction), efficiencies for each gene were 101 ± 3% and 102 ± 4% for *PK* and *CYTB*, and respectively, 98.6% for *ND1* and 101% for *ND4*. The qRT-PCR was performed in a 96-well PCR plate with TaqMan Universal PCR Mastermix (Applied Biosystems cat no: 4364338) with 400 nM of each primer, 80 nM of fluorogenic Roche Universal Library probe and 5 µl of 3.13 ng total of template DNA per reaction. Amplification was performed using the default cycling parameters of 2 min at 50°C, 10 min at 95°C, and 40 cycles of 15 s at 95°C and 60 s at 60°C. The mean cycle time obtained by double derivatives (CalqPlex algorithm; Eppendorf, Westbury, NY) was designated as Ct. Relative mtDNA/nDNA was assessed by comparative Ct method, using the following equation: $mtDNA/nDNA = 2^{-\Delta Ct}$, where $\Delta Ct = Ct_{mitochondrial} - Ct_{nuclear}$. Each gene was analyzed in triplicates in which the CV% <1.

**Mitochondrial bioenergetics-** Briefly, an aliquot (0.5-1.0 x $10^6$) of lymphocytes or (2.0 x $10^6$) fibroblasts was added to the oxygen chamber in 0.3 ml of a buffer containing 0.22 M sucrose, 50 mM KCl, 1 mM EDTA, 10 mM $KH_2PO_4$, and 10 mM HEPES, pH 7.4. Oxygen consumption rates were evaluated in the presence of (i) 1 mM ADP plus 1 mM malate-10 mM glutamate followed by the addition of 5 µM rotenone; (ii) 10 mM succinate followed by the addition of 1 mM malonate and 3.6 µM antimycin A; and (iii) 10 mM ascorbate and 0.2 mM *N,N,N’,N’*-tetramethyl-p-phenylenediamine followed by the addition of 1 mM KCN. The activities of mitochondrial succinate oxidase and cytochrome *c* oxidase were evaluated as the difference of oxygen uptake recorded before and after the addition of the inhibitors malonate and KCN, respectively. The respiratory control ratio (RCRu) was calculated as the ratio between oxygen uptake rates of intact cells supplemented with 10 mM glucose (present in RPMI-1640) in State 3u (with 2 µM carbonylcyanide-*p*-trifluoromethoxyphenylhydrazone; FCCP) and State 4 (with 0.2 µM oligomycin; (Napoli et al., 2013a). SRC (spare respiratory capacity) was calculated as the ratio (in percentage) between State 3u and basal respiration, whereas ROS/proton leak was calculated as the ratio (in percentage) between State 4 and basal respiration. Protein concentration was determined by the Lowry method and calculated according to bovine serum albumin standard curve.

**Evaluation of mitochondrial mass, morphology and distribution by confocal microscopy in fibroblasts-** Cells were seeded on sterile coverslips, grown over night at 37°C and then incubated for 30 min at 37°C with 0.5 µM MitoTracker Red CMXRos (MolecularProbes Inc., Eugene, OR, USA) diluted in growth media. After staining, cells were washed with media and fixed in 3.7% formaldehyde for 10 min. Fixed cells were then washed in PBS and blocked/permeabilized for 30 min in blocking buffer at 20-22˚C, counterstained with 1 µg/ml 4′ ,6 diamidino-2-phenylindole (DAPI) and mounted on glass slides with ProLong Gold anti-fade mountant for fixed cells (ThermoFisher Scientific, Waltham, MA). Fluorescent images (10-15 for each cell line) were obtained using an Olympus FV1000 laser scanning confocal microscope (excitation and emission

wavelengths 594 and 660 nm) at 60× magnification.

**Western blotting conditions-** Fibroblasts were lysed in RIPA buffer. After a brief centrifugation at 13,000 x *g* to eliminate cell debris, 25 µg of proteins were solubilized in SDS sample buffer (Thermo Fisher Scientific) and loaded onto a 4-12% bis-tris gel (Thermo Fisher Scientific). After transfer with an iBlot apparatus (Thermo Fisher Scientific), membranes were blocked with LI-COR blocking buffer (LI-COR Biosciences, Lincoln, NE) for 1 h at 20-22˚C and subsequently probed over night at 4°C with the antibodies reported in **Supplementary Table 1**. In some instances, membranes were probed with anti-β-actin antibody as loading control for 1 h at 20-22˚C. Similarly, incubation with goat anti-rabbit and goat anti-mouse secondary antibodies was carried out for 1 h at 20-22˚C.

## Supplementary Tables

**Supplementary Table 1 List of antibodies, commercial sources and dilutions employed**

| Antibody | Species | Source | Dilution |
|---|---|---|---|
| ***Target for primary antibody*** | | | |
| β-actin | Mouse | Sigma | 1:20,000 |
| FMRP | Mouse | Sigma | 1:1,000 |
| GFER | Rabbit | Proteintech | 1:1,000 |
| HIF-1α | Rabbit | Cell Signaling | 1:1,000 |
| HUMMR | Rabbit | Proteintech | 1:1,000 |
| MIA40 | Rabbit | Proteintech | 1:1,000 |
| MnSOD | Rabbit | EMD Millipore | 1:1,000 |
| NDUFA9 | Rabbit | Proteintech | 1:1,000 |
| NDUFB7 | Rabbit | Proteintech | 1:1,000 |
| phospho-FOXO3a | Rabbit | Cell Signaling | 1:1,000 |
| FOXO3a | Rabbit | Cell Signaling | 1:1,000 |
| SCO2 | Rabbit | Cell Signaling | 1:1,000 |
| SIRT1 | Mouse | Cell Signaling | 1:1,000 |
| SIRT3 | Rabbit | Cell Signaling | 1:1,000 |
| Tubulin | Mouse | Cell Signaling | 1:1,000 |
| VDAC | Rabbit | Proteintech | 1:1,000 |
| ***Secondary antibody*** | | | |
| IRDye 800CW anti-Rabbit IgG | Goat | LI-COR | 1:10,000 |
| IRDye 680RD anti-Mouse IgG | Goat | LI-COR | 1:10,000 |

**Supplementary Figures**

**Supplementary Figure 1**

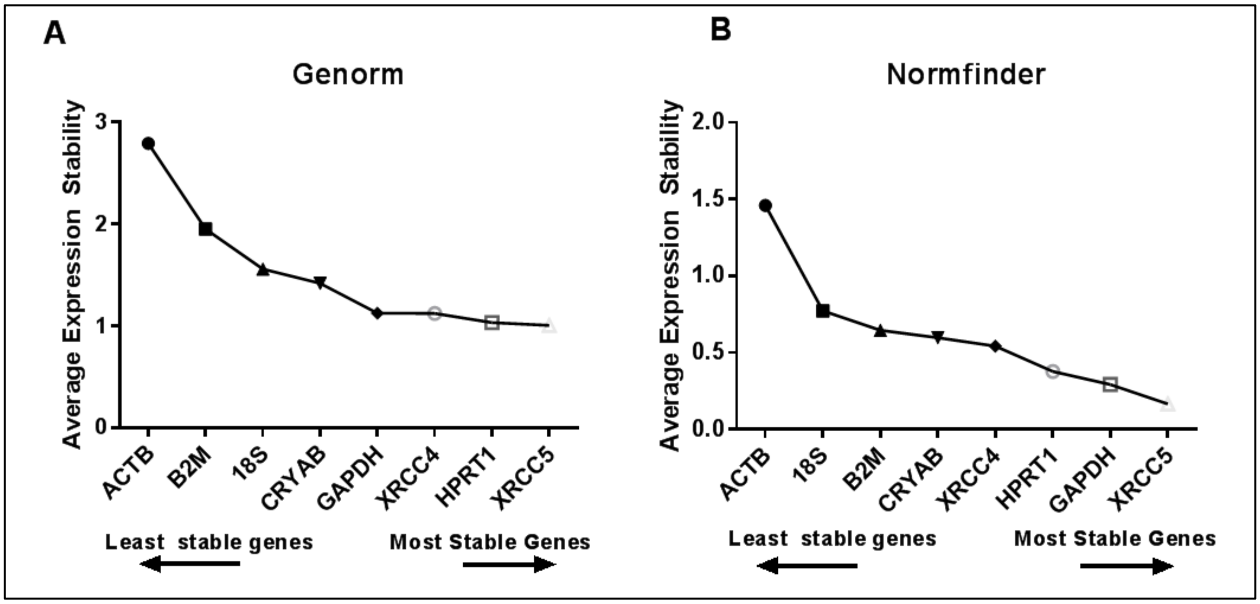


**Ranking of Gene Expression Stability in geNorm and NormFinder**
Most stable housekeeping genes for normalization were identified using the underlying principles and formulas behind geNorm and Normfinder.

**Supplementary Figure 2**

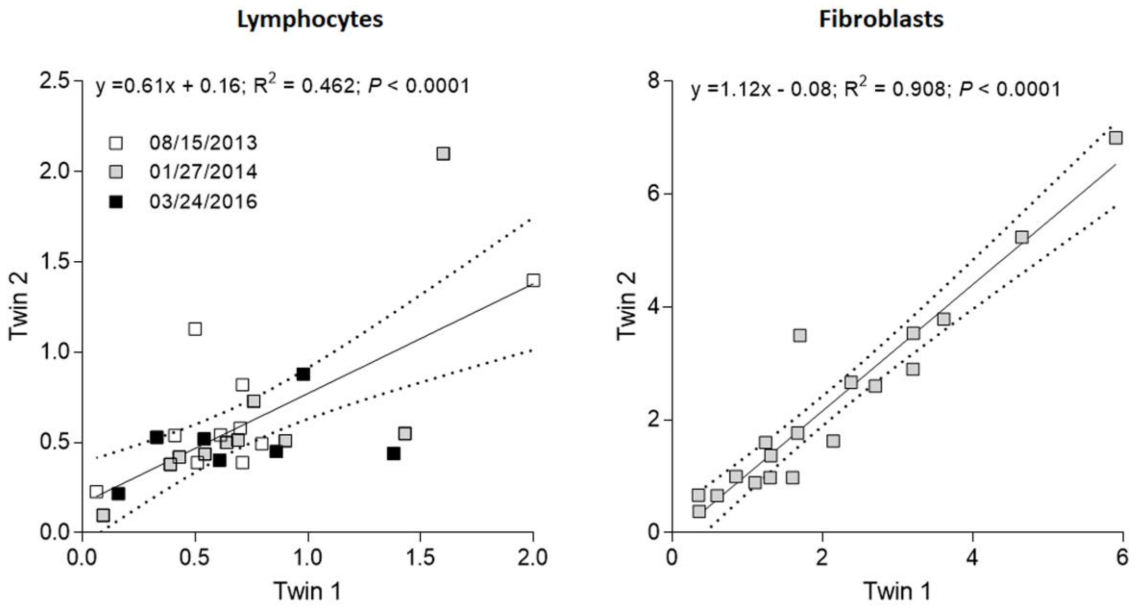


**Correlation of mitochondrial outcomes in lymphocytes and fibroblasts from twins**
Outcomes evaluated in lymphocytes (assessed at three different time points) and fibroblasts (evaluated on 01/27/2014) from twin 1 were plotted vs. the same outcomes in twin 2 (see Tables 3 and 4). The linear regression is shown as a solid line whereas the 95% CI is presented with dotted lines. Outcomes outside of the 95% CI in lymphocytes included: NAD- and FAD-linked oxygen uptake, CCO and RCRu. The only outcome outside of the 95% CI in fibroblasts was CCO.

## Supplementary Figure 3

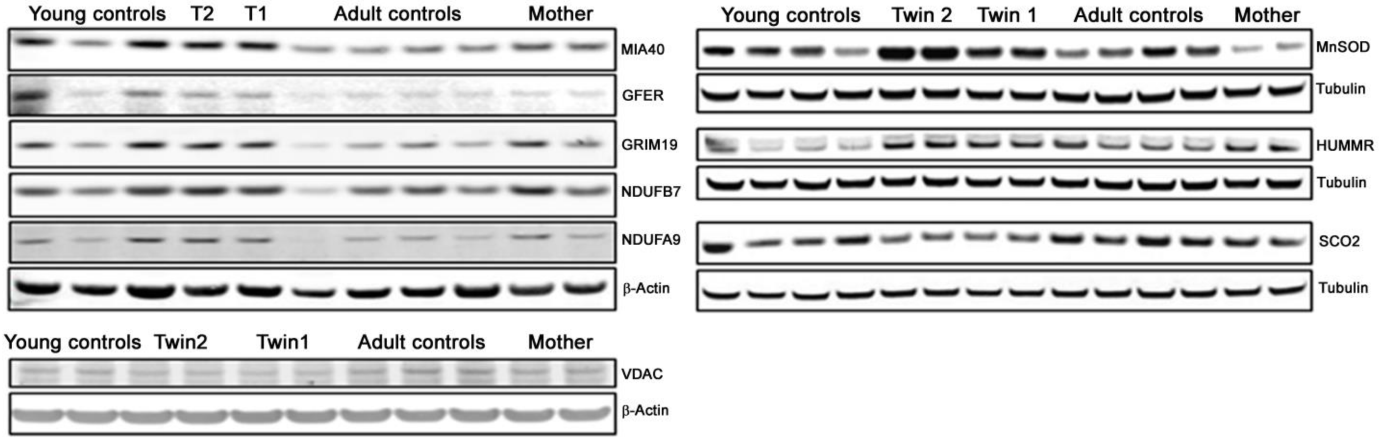


| Mitochondrial compartment | Outcome | Young controls Mean ± SEM [95%CI] | Twin 1 | Twin 2 | Adult controls Mean ± SEM [95%CI] | Mother |
|---|---|---|---|---|---|---|
| OM | HUMMR | 0.31 ± 0.08 [0.07-0.54] | 0.74 ± 0.01 | 0.76 ± 0.02 | 0.52 ± 0.07 [0.3-0.7] | 0.63 ± 0.07 |
| OM | VDAC | 0.78 ± 0.01 [0.76-0.79] | **0.72 ± 0.05** | **0.66 ± 0.03** | 0.94 ± 0.04 [0.87-1.00] | 0.91 ± 0.06 |
| IMS | MIA40 | 0.46 ± 0.04 [0.35-0.57] | 0.62 ± 0.01 | 0.68 ± 0.01 | 0.38 ± 0.01 [0.34-0.42] | 0.51 ± 0.01 |
| IMS | GFER | 0.4 ± 0.1 [0.1-0.7] | 0.31± 0.01 | 0.39 ± 0.03 | 0.29 ± 0.03 [0.22-0.36] | **0.18 ± 0.05** |
| IM | GRIM19 | 0.35 ± 0.03 [0.27-0.43] | 0.45 ± 0.01 | 0.55 ± 0.01 | 0.31 ± 0.02 [0.24-0.37] | 0.47 ± 0.01 |
| IM | NDUFB7 | 0.55 ± 0.02 [0.50-0.59] | 0.71 ± 0.04 | 0.90 ± 0.06 | 0.56 ± 0.08 [0.37-0.76] | 0.73 ± 0.04 |
| IM | NDUFA9 | 0.48 ± 0.04 [0.37-0.59] | 0.52 ± 0.06 | 0.65 ± 0.07 | 0.52 ± 0.05 [0.38-0.66] | 0.55 ± 0.08 |
| IM | SCO2 | 0.73 ± 0.08 [0.5-1.0] | 0.48 ± 0.02 | **0.435 ± 0.002** | 0.76 ± 0.06 [0.6-1.0] | 0.66 ± 0.05 |
| M | MnSOD | 0.6 ± 0.1 [0.3-1.0] | 1.00 ± 0.02 | 1.30 ± 0.02 | 0.61 ± 0.08 [0.4-0.9] | **0.21 ± 0.04** |

**Expression levels of selected mitochondrial proteins in fibroblasts from carriers**

Representative images and densitometry of Western blots from fibroblasts of young and adult controls, twins and their mother. Thirty-five µg of proteins were loaded *per* lane. Other methodological details are included in the Methods section. Data are shown as mean ± SEM for controls whereas the mean ± SD was used for the twins and their mother as an index of intra-experimental variability. Between brackets, the 95% CI obtained with control values. Bolded values are below the 95% CI; underlined ones are above the 95%CI. OM, IMS, IM, M refer to mitochondrial subcompartments, namely outer membrane, inter-membrane space, inner membrane, and matrix.

## References

Andersen, C.L., Jensen, J.L.Orntoft, T.F. (2004). Normalization of real-time quantitative reverse transcription-PCR data: a model-based variance estimation approach to identify genes suited for normalization, applied to bladder and colon cancer data sets. Cancer Res 64**,** 5245-5250. doi: 10.1158/0008-5472.CAN-04-0496.

Giulivi, C., Zhang, Y.F., Omanska-Klusek, A., Ross-Inta, C., Wong, S., Hertz-Picciotto, I., Tassone, F.Pessah, I.N. (2010). Mitochondrial dysfunction in autism. JAMA 304**,** 2389-2396. doi: 10.1001/jama.2010.1706.

Napoli, E., Hung, C., Wong, S.Giulivi, C. (2013a). Toxicity of the flame-retardant BDE-49 on brain mitochondria and neuronal progenitor striatal cells enhanced by a PTEN-deficient background. Toxicol Sci 132**,** 196-210. doi: 10.1093/toxsci/kfs339.

Napoli, E., Wong, S.Giulivi, C. (2013b). Evidence of reactive oxygen species-mediated damage to mitochondrial DNA in children with typical autism. Mol Autism 4**,** 2. doi: 10.1186/2040-2392-4-2.

Vandesompele, J., De Preter, K., Pattyn, F., Poppe, B., Van Roy, N., De Paepe, A.Speleman, F. (2002). Accurate normalization of real-time quantitative RT-PCR data by geometric averaging of multiple internal control genes. Genome Biol 3**,** RESEARCH0034.
